# A primer on cancer-associated fibroblast mechanics and immunosuppressive ability

**DOI:** 10.37349/etat.2023.00120

**Published:** 2023-02-20

**Authors:** Vanessa C. Talayero, Miguel Vicente-Manzanares

**Affiliations:** Molecular Mechanisms Program, Centro de Investigación del Cáncer and Instituto de Biología Molecular y Celular del Cáncer, Consejo Superior de Investigaciones Científicas (CSIC)-Universidad de Salamanca, 37007 Salamanca, Spain; Université Paris-Saclay, France

**Keywords:** Fibroblast, transforming growth factor-β, immunosuppression, cell mechanics, solid tumors, contractility

## Abstract

Cancer-associated fibroblasts (CAFs) are a major point of interest in modern oncology. Their interest resides in their ability to favor tumor growth without carrying genetic mutations. From a translational standpoint, they are potential therapeutic targets, particularly for hard-to-treat solid cancers. CAFs can be defined as non-tumor cells within the tumor microenvironment that have the morphological traits of fibroblasts, are negative for lineage-specific markers (e.g., leukocyte, endothelium), and enhance tumor progression in a multi-pronged manner. Two often-mentioned aspects of CAF biology are their ability to alter the mechanics and architecture of the tumor microenvironment, and also to drive local immunosuppression. These two aspects are the specific focus of this work, which also contains a brief summary of novel therapeutic interventions under study to normalize or eliminate CAFs from the tumor microenvironment.

## Introduction

In recent years, a lot of attention has been focused on the tumor microenvironment as an essential element of the cancer program. The non-malignant skewing of the function of bystander cells to promote tumor expansion is a crucial element of malignant transformation that nurtures the growth of cancer cells and provides physical and molecular components required for their proliferation and dissemination. Bystander cells also generate signals that further modify the genetic and biochemical makeup of actual cancer cells.

Fibroblasts are essential components of most tissue matrices. They are one of several cell types that populate most solid tissues, along with endothelial cells, pericytes, and diverse subclasses of myeloid and lymphoid cells, e.g., macrophages, dendritic cells (DCs), natural killer (NK) cells, NK T (NKT) cells, and T and B lymphocytes. Unlike these cell types, fibroblasts do not possess unique lineage-specific markers, therefore their phenotypic characterization is not straightforward [1]. Fibroblasts are widely believed to be the cell of origin of some types of sarcoma, that is, cancers of mesenchymal tissues [2].

In the context of carcinoma (solid tumors in which the cell of origin is of epithelial lineage), fibroblasts seldom acquire transforming mutations. However, carcinoma cells can alter the transcriptional, proteomic, and mechanical profiles of bystander fibroblasts to favor tumor cell growth and dissemination. Interestingly, this seems to depend on the degree of transformation of the carcinoma cells. A recent study showed that highly dedifferentiated, mesenchymal colon carcinoma cells trigger fibroblast conversion into cancer-associated fibroblasts (CAFs), whereas less dedifferentiated, epithelial cells of the same origin did not promote CAF transformation [3]. These effects depend on various types of stimuli, most notably cytokines, e.g., transforming growth factor-β (TGF-β). In this regard, an early study showed that TGF-β increased smooth muscle actin (SMA) expression in various types of fibroblasts [4]. Such cancer-altered fibroblasts are commonly referred to as CAFs.

CAFs are crucial players in the tumor microenvironment as they promote tumor cell survival, proliferation, and dissemination. They do so by various mechanisms, including secretion of multiple cytokines, e.g., TGF-β, leukemia inhibitory factor (LIF), growth-arrest specific (GAS) proteins, fibroblast growth factor (FGF), or hepatocyte growth factor (HGF) [5]. In addition, they stimulate tumor growth by triggering angiogenesis [6]. This work focuses on the ability of CAFs to promote tumor cell survival by contributing to local immunosuppression and to boost cancer cell propagation and dissemination by modifying the mechanics of the primary tumor microenvironment to enhance proliferation and facilitate individual or collective cell migration. In addition, CAFs contribute to invasion and metastasis directly by tugging tumor cells out of the primary mass.

## Possible origins and main features of CAFs

It is unlikely CAFs have a single origin. CAFs may evolve from pre-existent, resident fibroblasts embedded into the tissue. However, other cell types can be driven towards a CAF or CAF-like phenotype through epithelial-mesenchymal transition (EMT)-like processes, e.g., endothelial cells or pericytes [7, 8]. Another possible source is immature hematopoietic progenitors attracted by the transformation process that directly differentiate into CAFs [9]. Whether diverse tumors bear specific proportions of CAFs of different origins has yet to be examined in depth.

Multiple subclasses of CAF can be defined based on transcriptomic/proteomic alterations (for example, see [10]). Phenotypic/functional CAF cataloging is a very active field of research, and a definitive consensus has yet to be reached [5]. However, there are common traits that can be used to separate CAFs from normal fibroblasts. Briefly, most CAFs display elevated levels of fibroblast-associated protein (FAP), smooth muscle actin [SMA, gene ACTin isoform alpha 2 (*ACTA2*), platelet-derived growth factor (PDGF) receptors, vimentin, S100A4, podoplanin (PDPN) and other proteins and microRNA. Our work does not aim to provide a rigorous, or comprehensive, definition of CAF markers, mainly because there is a high degree of heterogeneity that makes general categorization almost impossible. In addition, recent efforts in classifying CAFs according to their expression profile have been reviewed elsewhere [11, 12]. In addition, CAFs from various origins have different levels of the markers described above. Also, the fact that CAFs are embedded in the tumor microenvironment means that they are subject to continuous transcriptomic and proteomic revision, leading to a high degree of heterogeneity [13].

## The “malignant” nature of CAFs

CAFs are not malignant per se. In fact, CAF is not that dissimilar from fibrotic fibroblasts that contribute to the healing of wounds, or smooth muscle cells that control sphincter function or blood pressure. However, they can exert profound modifications on the tumor microenvironment, contributing to the extension of the cancerous lesion and promoting the dissemination of tumor cells. In addition, CAFs impair the immune response through several mechanisms, mainly local production of TGF-β. Clinically, high numbers of CAF, or the local presence of CAF markers (mainly SMA, but also other markers, e.g., FAP or S100A4) [14–20], correlate with poor prognosis in different types of tumors. This is the conceptual basis of the identification of CAFs as potential therapeutic targets (see section “Clinical approaches to normalize the mechanical alterations and immunosuppression induced by the fibroblast-to-CAF transition”).

## CAFs stiffen the matrix, triggering pro-cancer mechanical and biochemical modifications to the tumor microenvironment

CAFs are more contractile than normal fibroblasts [21]. The main reason is the elevated expression of SMA compared to normal fibroblasts. This is a general feature of most CAFs [11]. It follows that SMA can be considered a marker of tissue malignization in non-fibrotic areas, even if fibroblasts themselves are not malignant per se. Increased SMA expression is sufficient to increase the ability of the cell to contract. The reason for this remains unclear. This is due to the fact that specific roles for actin isoforms are much less characterized than those of the other major component of the contractile apparatus, myosin II. An interesting hypothesis is that SMA (which belongs to the α-actin subfamily) preferentially associates with myosin II and drives contraction [22]; whereas non-muscle, β-actin tends to accumulate in the cortex and protrusive regions (where myosin II is much less abundant), becoming a lesser player in contraction [23]. While there is no biochemical demonstration of this hypothesis to date, several pieces of evidence indicate that such isoform-dependent functional segregation may be a part of the puzzle. For one, the induction of one actin isoform may repress the others. Hence, it is possible that CAFs containing elevated levels of SMA (α2-actin) display lower levels of β- and/or γ-actin. In addition, ACTA2^−/−^ mice remain viable, despite showing defects in vascular contractility and control of blood pressure [24]. This means that other actin isoforms can compensate for the deletion of the smooth muscle-specific isoform except in very specific cell types, such as blood vessel-lining smooth muscle cells, where SMA is the predominant isoform.

**Figure 1.**
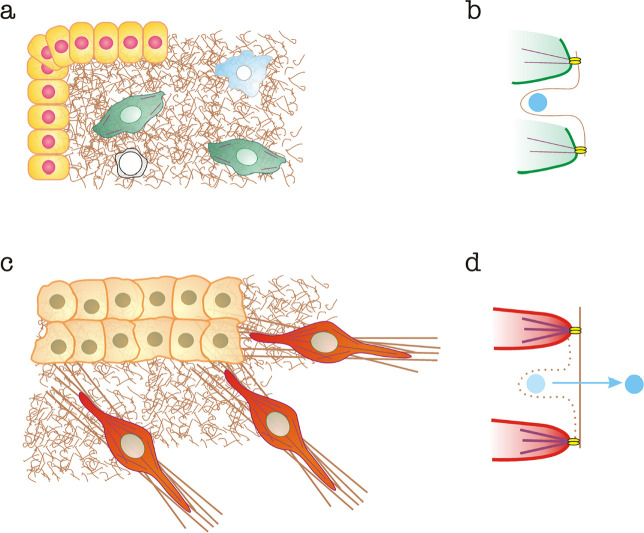
Alterations to the organization of the ECM and mechanical release of matrix-bound TGF-β mediated by CAFs. a) Normal tissue. Epithelial cells are shown in yellow, normal fibroblasts in green, macrophages in blue and T cells in white. Normal matrices are represented as brown “curly” fibers; b) curly fibers contain cryptic sites that bind TGF-β (shown in light blue) preventing its interaction with membrane-bound TGF-β receptors. Interaction with fibroblast integrins (bright yellow) linked to normal actin bundles (in purple) does not exert sufficient force to destroy the cryptic binding site and release TGF-β; c) tumor microenvironment, containing a carcinoma mass (light orange) and CAFs (red). Some of the matrix is bundled (brown straight lines); d) CAF integrins linked to SMA-enhanced bundles pull on the matrix, stretching it and eliminating cryptic binding sites, thereby releasing TGF-β, which is free to interact with TGF-β receptors

Increased CAF contractility enhances their ability to remodel fibrous matrix components, e.g., fibronectin or collagen [21], leading to increased numbers of large bundles of extracellular matrix (ECM) component fibers [1, 25]. Increased matrix bundling has multiple consequences. Cells proliferate better in rigid substrates and microenvironments [26]. Likewise, tumor cells migrate better along over-bundled matrices [27]. In addition, matrix stiffening promotes the release of TGF-β, which remains bound to collagen matrices [28]. This is a force-dependent mechanism in which integrins transmit cellular contractility to the matrix, bringing matrix filaments together into long, tense bundles (Figure 1). Matrix-bound TGF-β is much more unlikely to interact with plasma membrane-associated TGF-β receptors on cellular surfaces. Released TGF-β has multiple effects on the tumor microenvironment. In addition to its immunosuppressive effects (see section “CAFs trigger immunosuppression: a key role for TGF-β at the crossroads between mechanics and transcriptomics”), TGF-β drives fibroblasts to become CAFs [29] and promotes EMT in tumor cells and other cells of the tumor microenvironment, e.g., endothelial cells and pericytes [30]. In this manner, TGF-β constitutes a crucial switch that mediates the conversion of the mechanical modifications of the tumor microenvironment into potent biochemical signals that boost the cancer program in a local manner.

## CAF “locomotives” drive tumor cells toward egress sites to favor metastatic dissemination

In a landmark paper in 2006, Gaggioli and co-workers [31] showed that tumor fibroblasts acted as “locomotives” to pull chains of tumor cells out of tumor masses. This is not an exclusive property of CAFs, as tumor-associated macrophages (TAMs) can perform a similar function [32]. Also, this is not the only mechanism of tumor cell dissemination, as individual cancer cells can move in an autonomous manner using collagen fibers as durotactic tracks [33]. Whether “locomotive”-based (CAF or TAM) or individual cancer cell migration predominates is likely context-dependent. Some issues to consider include i) the degree of plasticity of tumor cells, e.g., the degree of mesenchymal transformation due to the implementation of EMT changes. Tumor cells that have undergone EMT are independent of CAF or TAM to migrate, as they down-regulate junctional molecules, thereby gaining independence from the main tumor mass; ii) the number and nature of available “locomotives”. Some tumors may contain low numbers of CAFs and/or TAMs, thus making “locomotive”-based chain migration more unlikely; iii) the types of contacts between cells. Neighboring tumor cells are likely at similar stages of differentiation. For example, poorly dedifferentiated carcinoma cells express very low levels of surface E-cadherin [34]. However, they still express other cell-cell adhesion molecules such as N-cadherin [35], which may contribute to the maintenance of stable cell-cell contacts and preserving a certain degree of mass integrity so they can be pulled out as chains by CAF/TAM “locomotives”. Conversely, more plastic cells may have heterogeneous levels of cadherins, creating “weak links” that may break down and favor individual migration.

Regarding cell-cell contacts, a major issue is how “locomotives” establish stable contacts with follower tumor cells. CAFs express very low levels of E-cadherin [36]. However, they do express N-cadherin [36], similar to tumor cells, in which N-cadherin expression correlates with increased motility [35–37]. Hence, N-cadherin homotypic interactions are the most likely mode of interaction between CAF leader and cancer follower cells. Another possibility is the existence of heterotypic N-cadherin/E-cadherin contacts. In this regard, Trepat and co-workers [38] showed that CAF and follower tumor cells display heterotypic clusters of N-cadherin (on the CAF side) and E-cadherin (on the tumor cell side) at cell-cell contacts. The biochemical details of these heterotypic contacts, or whether they contribute significantly to the interaction between the CAF/TAM leader cell and the first tumor follower cell together remains unaddressed.

**Figure 2.**
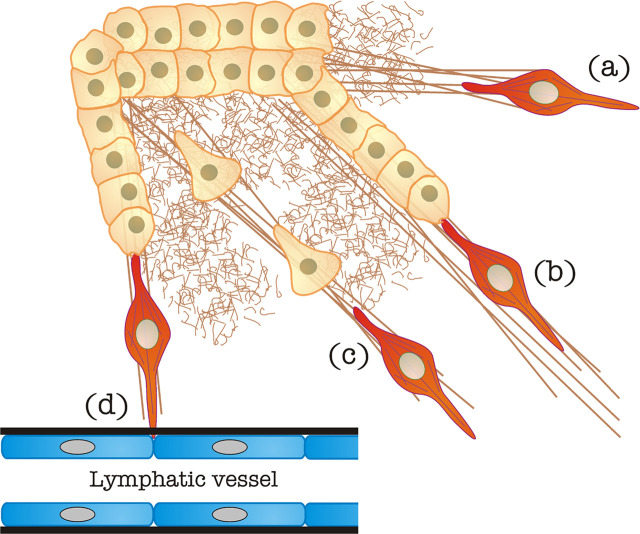
CAF-mediated egress of tumor cells from the primary mass. (a) Matrix stiffening caused by CAFs, as also shown in Figure 1; (b) “locomotive”-based chain migration, in which CAFs (in red, it may also represent a TAM, see section “CAF “locomotives” drive tumor cells toward egress sites to favor metastatic dissemination” for details) drag chains of tumor cells along matrix bundles; (c) matrix stiffened by CAFs can be used by individual cancer cells to undergo mesenchymal migration; (d) CAFs can degrade the basement membrane (in black) of blood/lymphatic vessels to favor tumor cell intravasation

Dissemination to distant organs occurs through one of the two major circulatory systems, blood or lymphatic vessels. A major question in the field is whether tumor cells egressing the primary tumor, tugged or not by CAFs or TAMs, migrate directionally toward entry points in lymphatic or blood vessels, adjacent to or inside the tumor. Whereas directional migration is a more interesting possibility from an intellectual standpoint, the fact remains that most tumor cells and CAFs do not express chemokine receptor 7 (CCR7), which is the main guidance receptor used by cells that migrate directionally towards lymphatic egress sites, such as DCs [39]. Therefore, it is more likely that tumor cells, guided or not by cellular “locomotives”, migrate in a random manner radiating outwards from the primary tumor’s core. Some of them are due to find blood or lymphatic vessels to pass onto circulation and undergo metastasis (Figure 2). The vessel basement membrane is likely a strong barrier that opposes intravasation. Cancer cell chains led by CAFs may take advantage of the highly degradative capability of the latter (Figure 2). Another possibility is the co-option of other chemokine/CCR pairs. One example is CCR5, which is expressed by a number of solid tumors and could steer tumor cells toward C-C chemokine ligand 5 (CCL5)-expressing endothelial cells (reviewed in [40]). Other chemo-, hapto- or durotactic gradients may also steer individual and chain tumor cell migration.

## CAFs trigger immunosuppression: a key role for TGF-β at the crossroads between mechanics and transcriptomics

TGF-β causes immunosuppression by acting on macrophages, tilting their function to favor tumor growth, and promoting their ability to release other immunosuppressive cytokines, such as interleukin-10 (IL-10) [41, 42]; it also inhibits DC function [43], curbs T cell responses [44] and inhibits NK cells [45–47]. CAFs directly increase the local concentration of TGF-β by expressing it [48], and indirectly by promoting the release of matrix-associated TGF-β through enhanced contractility (see section “CAFs stiffen the matrix, triggering pro-cancer mechanical and biochemical modifications to the tumor microenvironment” and Figure 1), which increases the actual local concentration of TGF-β available. It can be argued that TGF-β is a crucial element that transforms the mechanical and biochemical modifications of the CAF conversion process into a powerful immunosuppressive signal that inhibits innate immune responses, e.g., macrophage-mediated phagocytosis of transformed cells. TGF-β also curbs adaptive immunity by inactivating DCs and T cell responses through different mechanisms, such as i) decreasing the number of T cells reactive against the tumor, as TGF-β decreases DC-mediated antigen capture and presentation to T cells in lymph nodes [43]; ii) curbing T cell proliferation; and iii) limiting T cell infiltration into the tumor.

## Clinical approaches to normalize the mechanical alterations and immunosuppression induced by the fibroblast-to-CAF transition

**Figure 3.**
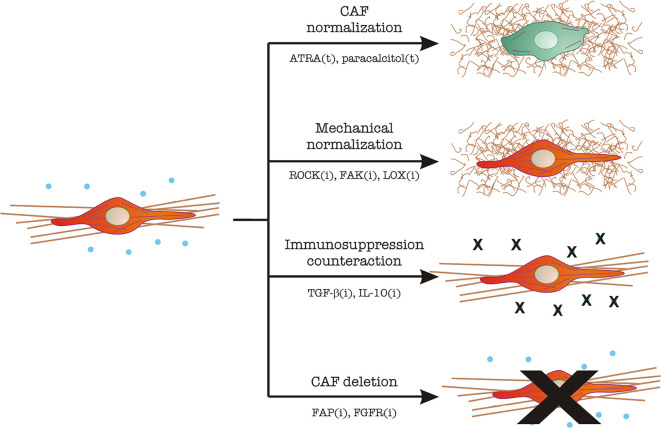
CAF targeting in tumor therapy. Image depicts the main mechanisms described under section “Clinical approaches to normalize the mechanical alterations and immunosuppression induced by the fibroblast-to-CAF transition”. CAFs are represented in red, with a straight and bundled matrix underneath. Immunosuppressive cytokines (TGF-β, IL-10) are shown as blue dots. Normal fibroblasts are represented in green, with fewer cytoskeletal filaments and a thin, curled matrix underneath. Strategies include CAF normalization, mechanical normalization, counteracting immunosuppression, and CAF inhibition/elimination. The main molecular players in each strategy are indicated. The letter “t” denotes treatment with the indicated molecule. The letter “i” denotes inhibitory strategies (antibodies, small molecule inhibitors, RNAi, etc.). ATRA: all-trans retinoic acid; ROCK: Rho-associated coiled-coil kinase; FAK: focal adhesion kinase; LOX: lysil oxidase; FGFR: FGF receptor

The various and important roles of CAFs in tumor growth and dissemination make them possible therapeutic targets, at least on paper. However, their heterogeneity and lack of specific markers have made it difficult to see progress in this regard. Recent clinical efforts to reduce the number of CAFs, or normalize their function, have been extensively reviewed [5]. These efforts can be roughly divided into several types (Figure 3):

(i) CAF “normalization”, which aims to decrease the features that make CAFs efficient helpers of the tumor program. Two examples include the use of ATRA or paracalcitol, which normalize the appearance of stellate cells [5].

(ii) Mechanical normalization, which aims to reduce contractility, theoretically decreasing tumor cell growth and dissemination. At the same time, these efforts would decrease the levels of free TGF-β by reducing mechanical-driven release (see section “CAFs stiffen the matrix, triggering pro-cancer mechanical and biochemical modifications to the tumor microenvironment”). These include:

 a. ROCK inhibitors. ROCK is a serine/threonine kinase that acts downstream of the small GTPase RhoA and triggers myosin II activation by inhibition of MYosin PhosphaTase 1 (MYPT1, reviewed in [49]). Therefore, ROCK inhibition should decrease contractility. In this context, ROCK targeting not only inhibits pancreatic cancer cell proliferation and increased chemotherapy efficiency, but also decreases collagen bundling, which is a sign of fibroblast inhibition [50].

 b. FAK inhibitors: FAK is a Tyr kinase that controls the downstream effects of integrin binding to the ECM. Signaling downstream of integrins have multiple effects in this context, mainly TGF-β activation [51] and matrix reorganization in a myosin II-dependent manner [52].

 c. LOX inhibitors: LOXs, or lysil oxidases, are extracellular oxidases that cross-link collagen, contributing to the formation and stabilization of large collagen bundles [53, 54]. LOX inhibition would decrease matrix bundling (and thus stiffening) [55], curbing tumor growth and TGF-β availability.

(iii) Inhibition of immunosuppressive effects: These efforts are aimed at inhibiting the immunosuppressive effects and directed mainly to TGF-β and IL-10 to a lesser extent. Different combinations of humanized monoclonal antibodies against TGF-β, TGF-β receptors, DC-based vaccines, and short RNA-based techniques are under study [56].

(iv) Inhibition of CAF function or direct CAF deletion: The idea underlying this approach is to reduce the number of CAFs in the tumor. Some CAF membrane receptors are being used as targets in these efforts, including FAP, PDGF, and FGF receptors (reviewed in [5]).

## Conclusions

CAFs remain remarkably uncharacterized, although a barrage of recent primary studies and reviews seems to indicate otherwise. Despite their heterogeneity and dynamic evolution within the tumor microenvironment, it seems that their boosting effect on the cancer program stems from their hyper-contractile nature, which reorganizes the matrix; and their immunosuppressive ability. Our intention here was to provide a glimpse into how these two properties are interconnected and illustrate the current state of the art. It is unlikely that CAF therapy is efficient on its own. However, it may become a crucial form of neo-adjuvant therapy that can limit tumor growth and complement chemotherapy, immunotherapy and/or other forms of targeted therapy.

## Abbreviations

ATRA: all-trans retinoic acid
CAFs: cancer-associated fibroblasts
CCR7: chemokine receptor 7
DCs: dendritic cells
ECM: extracellular matrix
EMT: epithelial-mesenchymal transition
FAK: focal adhesion kinase
FAP: fibroblast-associated protein
FGF: fibroblast growth factor
IL-10: interleukin-10
LOX: lysil oxidase
NK: natural killer
ROCK: Rho-associated coiled-coil kinase
SMA: smooth muscle actin
TAMs: tumor-associated macrophages
TGF-β: transforming growth factor-β

## Declarations

### Copyright

© The Author(s) 2023.
